# The Clinical Application Value of Cytokines in Treating Infectious Diseases

**DOI:** 10.1371/journal.pone.0098745

**Published:** 2014-06-02

**Authors:** Qing Ye, Wen-Xia Shao, Xiao-Jun Xu, Ying-zhi Yang

**Affiliations:** 1 Clinical Laboratory, The Children's Hospital of Zhejiang University School of Medicine, Hangzhou, China; 2 Clinical Laboratory, Hangzhou First People's Hospital, Hangzhou, China; 3 The Department of Hematology/Oncology, The Children's Hospital of Zhejiang University School of Medicine, Hangzhou, China; 4 Zhejiang University City College, Hangzhou, China; University of Cape Town, South Africa

## Abstract

We performed a prospective study to evaluate the abilities of inflammatory cytokines to rule out the potential risk of sepsis and intracranial infection and to estimate the function of inflammatory cytokines in discriminating Gram-negative bacteria from Gram-positive ones through ROC analysis. During the course of the study, Levels of serum inflammatory cytokines were measured by flow cytometry at the onset of diseases of patients who suffered from sepsis or intracranial infection. A total of 299 cases of sepsis and 43 cases of intracranial infection were observed during the study. It is noticed that there is no difference of inflammatory cytokine levels between sepsis group and intracranial infection group. The area under ROC curve (AUC) of cytokines, such as IL-2, IL-6 and IL-10 were 0.901, 0.86, 0.888, respectively, which was employed to rule out the diseases of sepsis and intracranial infection. Through comparisons with the patients who were infected by Gram-positive bacteria or Gram-negative ones, it is estimated that IL-6 and IL-10 sharply elevated in patients with Gram-negative bacteria infection (median levels, pg/mL: IL-6: 116.6 vs. 25.4, *P* = 0.000; IL-10: 13.7 vs. 6.3, *P* = 0.000). Additionally, IL-2 significantly decreased when patients suffered from Gram-negative bacteria infection (median levels, pg/mL: IL-2: 2.2 vs. 2.7, *P* = 0.031). The AUCs for detecting cytokines, including IL-2, IL-10 and LOGREGR.Pred_IL-2+IL-10 were 0.581 (95% CI, 0.526 to 0.634), 0.661 (95% CI, 0.608 to 0.712) and 0.735 (95% CI, 0.685 to 0.782), respectively, which was used to evaluate the function of inflammatory cytokines in discriminating Gram-negative bacteria from Gram-positive ones infection. This paper indicates that IL-2, IL-6 and IL-10 are effective biomarkers to rule out sepsis and intracranial infection. Additionally, the combination of IL-2 and IL-10 is an effective biomarkers to diagnose whether patients afflicted by Gram-negative bacteria.

## Introduction

Children with sepsis have a higher mortality than those without sepsis [1]. In Italy, in paediatric intensive care units severe sepsis and septic shock have a mortality rate of 17.7% and 50.8%, respectively [2]. A retrospective observational cohort dataset from seven U.S. states from 1995, 2000, and 2005 showed in 2005, 17,542 children were hospitalized with severe sepsis in the seven states; the data shows that the cases of pediatric severe sepsis has increased by 81% since 1995, and by 45% since 2000. Between 1995 and 2005, the prevalence of severe sepsis in newborns had more than doubled, from 4.5 to 9.7 cases per 1,000 births [3]. Therefore, early diagnosis and ealry treatment effectively prevent the newborns from death. Bacteria are the most common cause of severe sepsis in children [4], [5]. As a result of the low positive rate and long duration, microbiologic culture is not sensitive enough for the early detection of bacteria in febrile patients. Even though the results of suffering microbiological infection are positive, clinicians still need to be aware of whether the results are false or not, to preclude the mixed infection or of environmental contamination.

Th1/Th2 balance theory has been widely accepted in academia and confirmed. Th1/Th2 cytokines plays a very important role in anti-infection immunity, so we thought there would be changes of serum Th1/Th2 cytokine profiles and levels in patients with infection. In addition, the Th1/Th2 kit has been regularly used for many years in our laboratory and our experience indicates that this reagent is stable and the results obtained are consistent. So, we studied the clinical application value of Th1/Th2 cytokines in treating infectious diseases.

Additionally, a prospective study on the role of quick cytokine profiles examinated by flow cytometry aimed at pediatric hematology/oncology patients has been performed in our hospital, which shows that a series of cytokines are crucial for evaluating the severity of shock and the selection and/or timely withdrawal or switch of antibiotics [6]. However, that study was only performed among pediatric hematology/oncology patients. In this article, we attempt to employ the results in patients with sepsis and intracranial infection, but without aplastic anemia or cancer. C-reactive protein (CRP), a kind of classical systemic inflammatory marker, was detected in this study to evaluate the ability of cytokines to eliminate sepsis and intracranial infection and to distinguish gram-negative bacteria (GNB) infection from gram-positive bacteria (GPB) infection.

## Patients and Methods

### Patients and definitions

This was a prospectively observational study conducted from November 2011 through March 2014 in the Children's Hospital of Zhejiang University School of Medicine. It was approved by the medical ethics committee of the Children's Hospital of Zhejiang University School of Medicine. Informed written consent was obtained from guardians on the behalf of the minor/child participants involved the study. Patients who met the following criteria were enrolled: (1) age less than 18 years old; (2) diagnosed with intracranial infection [7] or sepsis. The criteria of sepsis were according to the international pediatric sepsis consensus [8]. (3) Bacterial bloodstream infection and cerebral spinal fluid infection were documented by microbiological culture at the onset of sepsis or intracranial infection. In order to prevent unreliable results caused by contamination, the blood samples of each patient were taken at two different sites at the onset of sepsis. When coagulase-negative staphylococci were reported, they were considered as pathogens only when one of the following criteria was met: (a) the two samples from two different sites showed the same result; (b) if the other sample was negative, a PCR assay of the blood sample was positive for gram-positive bacteria; (c) same result from a repeated culture.

To assess the performance of the prediction power of cytokines in separate cohorts, Patients experiencing intracranial infection or sepsis between November 2011 and November 2013 were assigned to the derivation cohort, whereas those developing intracranial infection or sepsis from December 2013 through March 2014 were assigned to the validation cohort.

At the onset of sepsis or intracranial infection, blood samples or cerebrospinal fluid specimens were taken for microbiological analyses and for serum Th1/Th2 cytokine and CRP determination immediately. Chest radiography, CT scan, and abdominal ultrasound were performed to check the sites of infection if necessary.

### Cytokine and CRP determination

The blood samples were centrifuged at 1,000 g at 20°C for 20 min after clotting. The serum was carefully harvested and underwent the Th1/Th2 cytokine measurement by 320 flow cytometry immediately. Concentrations of IL-2, IL-4, IL-6, IL-10, tumor necrosis factor (TNF)-a, and interferon (IFN)-γ were quantitatively determined by the CBA kit–BDTM CBA Human Th1/Th2 Cytokine Kit II (BD Biosciences, San Jose, CA). Briefly, the CBA technique was based on six bead populations with distinct fluorescence intensities that had been coated with capture antibodies specific for IL-2, IL-4, IL-6, IL-10, TNF-α and IFN-γ proteins. The fluorescent dye had a maximal emission wavelength of approximately 650 nm (FL-3), which was detectable by flow cytometry. The cytokine capture beads were mixed with the phycoerythrin-conjugated detection antibodies and then incubated with recombinant standards or test samples to form sandwich complexes. Following the acquisition of sample data on a FACScaliburTM flow cytometer (Becton Dickinson, San Jose, CA, USA), the sample results were demonstrated in graphical and tabular format using the BD CBA Software (BD Biosciences, San Jose, CA, USA). The standard curve was set up for each individual set of reagents. The minimal and maximum limits of detection for all six cytokines were 1.0 and 5,000 pg/mL, respectively. Concentrations of CRP were measured by the QuikRead go instrument with QuikRead go CRP kits.

### Statistical analysis

The comparisons between the two groups were performed using the χ^2^ or Fisher's exact test for categorical variables and the Kruskal–Wallis H test (for three groups) or Mann–Whitney U test (for two groups) for continuous variables. Above statistical analyses was performed using SPSS Statistics18.0 software. P<0.05 was considered to be statistically significant. Receiver operating characteristic (ROC) curve was used to assess the diagnostic value of CRP, IL-2, IL-4, IL-6, IL-10, TNF-α and IFN-γ to rule out intracranial infection and sepsis, and to discriminate gram-negative from gram-positive bacteria infection by MedCalc 9.4.2.0 software. The comparisons of AUCs were performed using Z test. The optimal diagnosis threshold was determined according to Youden index J and relative sensitivity and specificity were calculated. Logistic regression was used to calculate predicted probabilities of various combinations of cytokines, and then the predicted probabilities were saved as a new indicator LOGREGR_Pred1 to assess the diagnostic value of different cytokines combination by ROC curve.

## Results

### Patients' characteristics

A total of 299 episodes of sepsis and 43 episodes of intracranial infection confirmed by microbiological culture were observed from November 2011 to November 2013. 299 episodes of sepsis occurred in 299 patients, with a median age of 1.75 years old (range, 0.03–17.5 years old) at the onset of sepsis and a male to female ratio of 2.52. The diagnoses of the underlying diseases were pneumonia (n = 152), sepsis (n = 85), enteritis (n = 16), bronchiolitis (n = 12), upper respiratory infection (n = 8), diarrhea (n = 8), tonsillitis (n = 8), bronchitis (n = 6) and jaundice (n = 4). Each episode of sepsis was categorized into either GPB or GNB. Of the 299 cases, GPB accounted for 75.3% of the episodes, with one main causative organism being coagulase-negative staphylococci. GNB accounted for 24.7% of the episodes, with two main causative organisms being Escherichia coli and serratia marcescens. 43 episodes of intracranial infection occurred in 43 patients, with a median age of 0.84 years old (range, 0.14–11.1 years old) at the onset of intracranial infection and a male to female ratio of 2.31. The diagnoses of the underlying diseases were encephalitis (n = 15), pneumonia (n = 10), purulent meningitis (n = 8), omphalitis (n = 3), and others (n = 7). Each episode of sepsis was categorized into either GPB or GNB. Of the 43 cases, GPB accounted for 72.1% of the episodes, with two main causative organisms being coagulase-negative staphylococci and micrococcus. GNB accounted for 27.9% of the episodes, with two main causative organisms being Escherichia coli and klebsiella pneumoniae. Thirty three healthy children without fever or any signs of infection were enrolled as the control cohort. The median age of the control group was 2.1 years old (range, 0.23–10.5 years old) with a male to female ratio of 1.5 (20/13). A total of 77 episodes of sepsis and 13 episodes of intracranial infection confirmed by microbiological culture were observed from December 2013 through March 2014. Detailed information is shown in table 1.

**Table 1:** Demographic characteristics of patients with blood stream infection or intracranial infection.

	Derivation cohort (n = 342)			Validation cohort (n = 90)	
Characteristics	blood stream infection (n = 299)	cerebral spinal fluid infection (n = 43)	*P* value	blood stream infection (n = 77)	cerebral spinal fluid infection (n = 13)
Median age (range), year	1.75 (0.03–17.5)	0.84 (0.14–11.1)	0.246	1.84 (0.11–16.0)	1.0 (0.10–10.3)
Gender (male/female)	214/85	30/13	0.857	56/21	9–4
Organism					
ram-negative bacteria	74 (24.7%)	12 (27.9%)		20 (26.0%)	4 (30.8%)
escherichia coli	30	4		12	2
serratia marcescens	9	0		2	0
pseudomonas aeruginosa	6	1		1	1
salmonella	5	0		0	0
pseudomonas maltophilia	5	0		1	0
ebsiella pneumoniae	4	4		3	1
acinetobacter baumannii	3	0		0	0
klebsiella oxytoca	3	1		0	0
haemophilus influenzae	2	1		0	0
moraxella	3	0		0	0
ochrobactrum anthropi	3	0		0	0
burkholderia cepacia	1	0		1	0
enterobacter cloacae	0	1		0	0
Gram-positive bacteria	225 (75.3%)	31 (72.1%)		57 (74.0%)	9 (69.2%)
coagulase negative staphylococcus	154	18		21	4
micrococcus	18	5		5	2
corynebacterium	10	0		6	0
enterococcus	8	2		8	0
streptococcus pneumoniae	8	3		4	1
bacillus	6	0		1	0
streptococcus mitis	6	1		3	1
staphylococcus aureus	5	0		4	0
nocardia asteroids	4	1		1	0
streptococcus viridans	3	0		2	0
streptococcus agalactiae	1	0		1	0
streptococcus salivarius	1	1		0	1
streptococcus bovis	1	0		1	0
underlying diseases					
pneumonia	152	10		29	3
sepsis	85	0		18	0
enteritis	16	0		9	0
bronchiolitis	12	0		7	0
upper respiratory infection	8	0		2	0
diarrhea	8	0		4	0
tonsillitis	8	0		4	0
bronchitis	6	0		3	0
jaundice	4	0		1	0
traumatic subdural haemorrhage	0	2		0	0
hydrocephaly	0	2		0	0
cerebral contusion	0	2		0	0
skull fracture	0	1		0	1
omphalitis	0	3		0	0
encephalitis	0	15		0	6
purulent meningitis	0	8		0	3

### Inflammatory cytokine levels and CRP in patients with sepsis and intracranial infection

The levels of six cytokines and CRP in the control group and patients with sepsis or intracranial infection are shown in Table 2. No difference of inflammatory cytokine levels was found between sepsis and intracranial infection groups, except CRP (median levels, mg/L: CRP: 10.0 vs. 39.0, *P* = 0.03). IL-4 did not elevated in patients with sepsis, but CRP and the inflammatory cytokine levels of IL-6, IL-10, TNF-α and IFN-γ significantly elevated when compared with the control group (median levels, mg/L: CRP: 10.0 vs. 2.0, *P* = 0.00; median levels, pg/mL: IL-6: 33.7 vs. 4.1, *P* = 0.00; IL-10: 6.9 vs. 2.4, *P* = 0.00; TNF-α: 2.5 vs. 2.3, *P* = 0.04; IFN-γ: 8.0 vs. 4.6, *P* = 0.00). IL-6, IL-10 and CRP in patients with intracranial infection were significantly elevated when compared with the control group (median levels, pg/mL: IL-6: 34.5 vs. 4.1, *P* = 0.00; IL-10: 9.2 vs. 2.4, *P* = 0.00; median levels, mg/L: CRP: 39.0 vs. 2.0, *P* = 0.00).

**Table 2:** Serum cytokine levels and CRP in the control group and patients with sepsis or intracranial infection.

parameters	sepsis (n = 299)	intracranial infection (n = 43)	control (n = 33)	P value^a^	P value^b^	P value^c^
IL-2 (pg/mL)	2.7 (1.0→20.7)	2.2(1.0→5.6)	5.8 (2.7→7.8)	0.14	0.00	0.00
IL-4 (pg/mL)	2.9(0.9→15.4)	2.45(1.1→4.7)	2.7 (1.1→4.0)	0.25	0.59	0.61
IL-6 (pg/mL)	33.7(1.6→1605.8)	34.5(1.2→1472.3)	4.1 (1.2→8.5)	0.57	0.00	0.00
IL-10 (pg/mL)	6.9(1.2→1783.2)	9.2(1.8→162.3)	2.4 (1.3→9.9)	0.60	0.00	0.00
TNF-α (pg/mL)	2.5(1.0→319.4)	2.6(1.0→25.2)	2.3 (1.1→3.1)	0.54	0.04	0.41
INF-γ (pg/mL)	8.0(1.4→788.6)	5.7(1.3→41.8)	4.6 (3.3→7.8)	0.05	0.00	0.12
CRP (mg/L)	10.0(1.0→160.0)	39.0(3.0→117.0)	2.0 (1.0→3.1)	0.03	0.00	0.00

a P value of sepsis group compare with intracranial infection group; ^b^P value of sepsis group compare with normal control group; ^c^P value of intracranial infection group compare with normal control group. Range and median values are represented for each group.

### ROC-analysis for the performances of inflammatory cytokine levels and CRP to rule out the possibility of sepsis and intracranial infection

**Figure 1:**
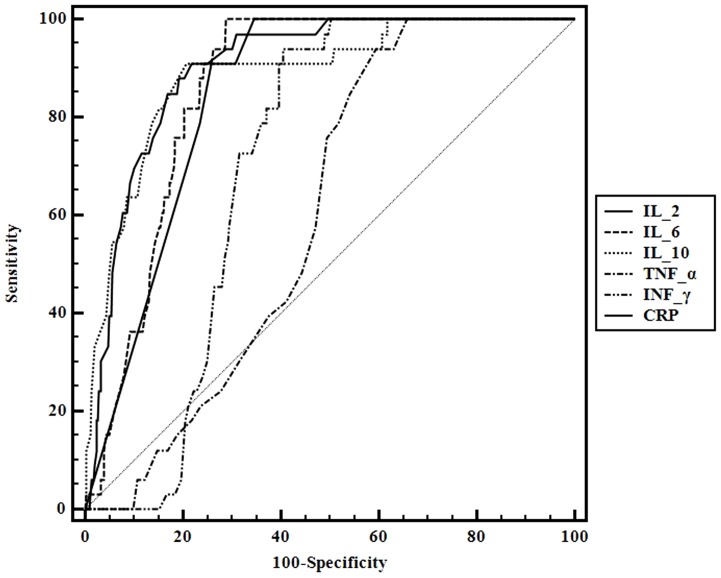
ROC curve for the performances of inflammatory cytokine levels and CRP to rule out the possibility of sepsis and intracranial infection.

As analyzed above, CRP and the three cytokines IL-2, IL-6 and IL-10 were differentially related to sepsis and intracranial infection. We evaluated the abilities of the four indicators to rule out the possibility of sepsis and intracranial infection by ROC analysis. The AUCs for IL-2, IL-6, IL-10 and CRP were 0.901 (95% CI, 0.866 to 0.930), 0.86 (95% CI, 0.821 to 0.894), 0.888 (95% CI, 0.851 to 0.918) and 0.848 (95% CI, 0.807 to 0.883) respectively, indicating that IL-2, IL-6 and IL-10 were effective biomarkers to rule out sepsis and intracranial infection. A serum IL-2 level greater than 4 pg/mL had a sensitivity of 90.9% and a specificity of 78.2% to rule out sepsis and intracranial infection (Youden index J: 0.6912); a serum IL-6 level equal to 8.5 pg/mL or lower had a sensitivity of 100.0% and a specificity of 71.0% to rule out sepsis and intracranial infection (Youden index J: 0.7104); a serum IL-10 level equal to 3.6 pg/mL or lower had a sensitivity of 90.9% and a specificity of 79.1% to rule out sepsis and intracranial infection (Youden index J: 0.7001). The AUCs for IL-2, IL-6, and IL-10 to rule out the possibility of sepsis and intracranial infection were bigger than CRP (Table 3, Figure 1).

**Table 3:** ROC-analysis for the performances of inflammatory cytokine levels and CRP to rule out the possibility of sepsis and intracranial infection.

diagnostic indicators	AUC	SE a	95% CI b	Youden index J	Associated criterion (pg/mL)	Sensitivity (%)	Specificity (%)	z statistic	Significance level
Derivation cohort (n = 342)									
IL-2	0.901	0.0208	0.866 to 0.930	0.6912	>4.0	90.9	78.2	1.903	0.0570*
IL-4	0.522	0.0437	0.470 to 0.574	0.1477	≤2.9	69.7	45.1	6.818	<0.0001*
IL-6	0.860	0.0203	0.821 to 0.894	0.7104	≤8.5	100.0	71.0	0.504	0.6144*
IL-10	0.888	0.0296	0.851 to 0.918	0.7001	≤3.6	90.9	79.1	1.128	0.2595*
TNF-α	0.604	0.0346	0.552 to 0.654	0.3454	≤2.7	93.9	40.6	6.139	<0.0001*
INF-γ	0.706	0.0264	0.656 to 0.752	0.5334	≤6.3	93.9	59.4	4.466	<0.0001*
CRP	0.848	0.0221	0.807 to 0.883	0.6548	≤4.0	100.0	65.5		
Validation cohort (n = 90)									
IL-2	0.890	0.0300	0.820 to 0.939		>4.0	90.9	75.6	1.661	0.0968*
IL-4	0.578	0.0525	0.485 to 0.666		≤2.9	69.7	55.6	3.768	0.0002*
IL-6	0.902	0.0264	0.835 to 0.948		≤8.5	100.0	73.3	2.571	0.0101*
IL-10	0.863	0.0358	0.789 to 0.918		≤3.6	90.9	77.8	1.077	0.2817*
TNF-α	0.708	0.0457	0.620 to 0.787		≤2.7	93.9	53.3	1.589	0.1121*
INF-γ	0.693	0.0455	0.604 to 0.773		≤6.3	93.9	56.6	2.006	0.0448*
CRP	0.807	0.0368	0.726 to 0.873		≤4.0	100.0	70.1		

a DeLong et al., 1988.

b Binomial exact.

*compared with CRP.

### Serum cytokine levels in patients infected with Gram-positive bacteria or Gram-negative bacteria

**Figure 2:**
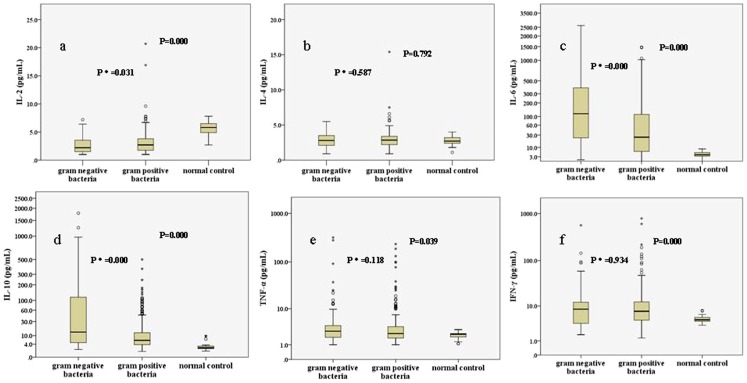
Serum cytokine levels in the normal control group and patients with gram-positive bacteria infection and gram-negative bacteria infection. The median levels of IL-2 in the three groups were respectively 5.8, 2.7, and 2.2 pg/mL; b IL-4, 2.7, 2.9, and 2.8 pg/mL; c IL-6, 4.1, 25.4, and 116.6 pg/mL; d IL-10, 2.4, 6.3, and 13.7 pg/mL; e TNF-α, 2.3, 2.4, and 2.8 pg/mL; f INF-γ, 4.6, 7.5, and 8.4 pg/mL. *P* indicated comparisons among three groups while p* indicated comparisons between gram-positive bacteria and gram-negative bacteria.

The levels of six cytokines in the control group and patients with Gram-positive bacteria or Gram-negative bacteria infection are shown in Figure 2. Comparisons among the three groups, IL-2, IL-6, IL-10, TNF-α and IFN-γ had statistical differences, but no difference was found on the serum level of IL-4. Comparisons between gram-positive bacteria and gram-negative bacteria infection, IL-6 and IL-10 sharply elevated in patients with gram-negative bacteria infection (median levels, pg/mL: IL-6: 116.6 vs. 25.4, *P* = 0.000; IL-10: 13.7 vs. 6.3, *P* = 0.000). In addition,IL-2 significantly decreased when patients suffered from Gram-negative bacteria infection (median levels, pg/mL: IL-2: 2.2 vs. 2.7, *P* = 0.031). However, serum levels of IL-4, TNF-α and IFN-γ were comparable (P>0.05).

### Establishment of model for GNB prediction

**Figure 3:**
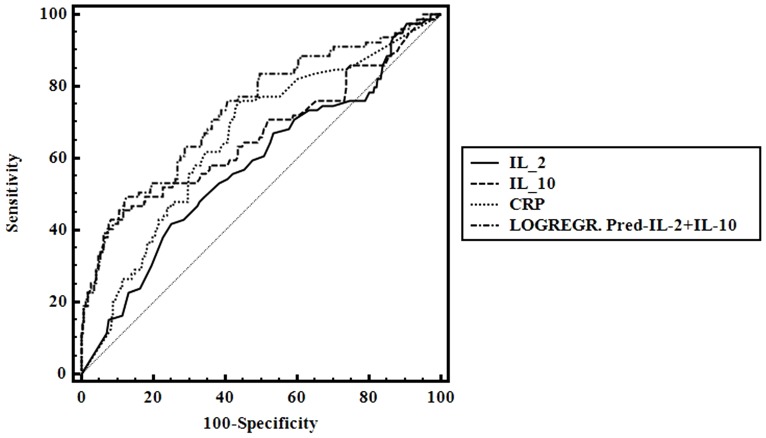
ROC curve for predictive value of cytokines for GNB in patients with sepsis or intracranial infection.

As analyzed above, IL-6 and IL-10 were sharply elevated in patients with gram-negative bacteria infection compared with gram-positive bacteria infection and IL-2 was significantly decreased. ROC curve was used to assess the diagnostic value of IL-2, IL-6, IL-10 and CRP to discriminate gram-negative from gram-positive bacteria by MedCalc 9.4.2.0. The AUCs for IL-2, IL-10, LOGREGR?Pred_IL-2+IL-10 and CRP were 0.581 (95% CI, 0.526 to 0.634), 0.661 (95% CI, 0.608 to 0.712), 0.735 (95% CI, 0.685 to 0.782), and 0.654 (95% CI, 0.600 to 0.705), respectively. The AUCs for LOGREGR?Pred_IL-2+IL-10 was bigger than IL-2 or IL-10 alone and its specificity was better indicating that the combination of IL-2 and IL-10 was a more effective biomarker in predicting GNB. In addition, its specificity in prediction of GNB infection was better than CRP (Table 4, Figure 3).

**Table 4:** Predictive value of cytokines for GNB in patients with sepsis or intracranial infection.

diagnostic indicators	AUC	SE a	95% CI b	Youden index J	Associated criterion (pg/mL)	Sensitivity (%)	Specificity (%)	Z statistic	Significance level
Derivation cohort (n = 342)									
IL-2	0.581	0.0379	0.526 to 0.634	0.1677	≤1.7	41.80	75.00	1.394	0.1633*
IL-10	0.661	0.0398	0.608 to 0.712	0.3483	>58.5	43.00	91.80	0.156	0.8764*
CRP	0.654	0.0352	0.600 to 0.705	0.3171	>11.0	74.70	57.00		
LOGREGR.Pred_IL-2+IL-10	0.735	0.0340	0.685 to 0.782	0.3687	IL-2<3.0 and IL-10>48.9	22.50	93.30	1.925	0.0542*
Validation cohort (n = 90)									
IL-2	0.568	0.0427	0.503 to 0.631		≤1.7	41.10	72.69	1.154	0.2486*
IL-10	0.650	0.0412	0.592 to 0.705		>58.5	42.47	90.74	0.495	0.6207*
CRP	0.615	0.0410	0.551 to 0.677		>11.0	72.58	50.56		
LOGREGR.Pred_IL-2+IL-10	0.711	0.0392	0.649 to 0.767		IL-2<3.0 and IL-10>48.9	18.52	96.83	1.902	0.0572*

a DeLong et al., 1988.

b Binomial exact.

*compared with CRP. LOGREGR?Pred_IL-2+IL-10 indicates the combination of IL-2 and IL-10.

### Validation of inflammatory cytokines to rule out the possibility of sepsis and intracranial infection in validation set

We then tested the performance of inflammatory cytokines to rule out the possibility of sepsis and intracranial infection in validation set. The AUCs for IL-2, IL-6, IL-10 and CRP were 0.890 (95% CI, 0.820 to 0.939), 0.902 (95% CI, 0.835 to 0.948), 0.863 (95% CI, 0.789 to 0.918) and 0.807 (95% CI, 0.726 to 0.873) respectively. A serum IL-2 level greater than 4 pg/mL had a sensitivity of 90.9% and a specificity of 75.6% to rule out sepsis and intracranial infection; a serum IL-6 level equal to 8.5 pg/mL or lower had a sensitivity of 100.0% and a specificity of 73.3% to rule out sepsis and intracranial infection; a serum IL-10 level equal to 3.6 pg/mL or lower had a sensitivity of 90.9% and a specificity of 77.8% to rule out sepsis and intracranial infection, which showed similar power to rule out the possibility of sepsis and intracranial infection to that in the derivation cohort (Table 3).

### Validation of cytokines for GNB prediction in validation set

We then tested the performance of cytokines for GNB prediction in validation set. The AUCs for IL-2, IL-10, LOGREGR?Pred_IL-2+IL-10 and CRP were 0.568 (95% CI, 0.503 to 0.631), 0.650 (95% CI, 0.592 to 0.705), 0.711 (95% CI, 0.649 to 0.767), and 0.615 (95% CI, 0.551 to 0.677), respectively. The AUC for LOGREGR?Pred_IL-2+IL-10 was bigger than IL-2 or IL-10 alone and its specificity was better indicating that the combination of IL-2 and IL-10 was a more effective biomarker in predicting GNB and its specificity in prediction of GNB infection were better than CRP, which showed similar power to predict GNB to that in the derivation cohort (Table 4).

## Discussion

Sepsis and intracranial infection are associated with uncontrolled and excessive cytokines release [9], [10], early diagnosis are crucial for patients' survival. We tried to detect cytokine levels of patients suffered from sepsis or intracranial infection. Concentrations of IL-2, IL-4, IL-6, IL-10, TNF-a and IFN-γ were quantitatively determined by the CBA kit–BDTM CBA Human Th1/Th2 Cytokine Kit II (BD Biosciences, San Jose, CA). No difference of inflammatory cytokine levels was found between sepsis and intracranial infection groups. Compared with the control group, the inflammatory cytokine levels of IL-2, IL-6 and IL-10 in patients with sepsis or intracranial infection were significantly elevated (both P<0.05). CD4+ T cell cytokines provide important regulatory and effector functions of T cells [9]. Among them, IL-2 plays a unique role. IL-2 is required for the generation and maintenance of regulatory T cells [10]. IL-2 is also required for fighting against infection and germs as research has shown that IL2_/_ mice themselves spontaneously develop multi-organ inflammation [9], [11]. IL-6 is a cytokine that can amplify acute inflammation [12], [13], stimulate the production of acute phase proteins [14] and induce leukocytosis and fever [15]. Also, IL-6 is able to contribute to the transition into the chronic phase of inflammation [16]. Interleukin-10 (IL-10) is a pleiotropic cytokine characterized by a broad spectrum of anti-inflammatory activities. IL-10 acts primarily on antigen-presenting cells inhibiting the release of proinflammatory cytokines and chemokines, overall limiting antigen-presenting cell function [17], [18]. IL-10 can also directly inhibit T-cell function and cytokine production [19], chemotaxis [20], and proliferation [21] to limit inflammation.

The existing studies have shown that T cell-suppression is one of the important factors of infection [22]. Treg-mediated IL-2 deprivation is a reason of T cell-suppression during infection [23]. Treg, such as Treg/CD39+, is one of these cells can efficiently suppress IL-2 expression of activated CD4+ T-cells [24]. Therefore, IL-2 often reduces when infection occurs. After the occurrence of infection, elevated IL-10 plays the role of anti-inflammatory. This is the potential mechanism for counter regulation of IL-2 versus IL-10 during sepsis or intracranial infection.

Based on the biological functions of IL 2, IL - 6 and IL - 10 in inflammatory reaction and abnormal expression of their serum levels observed by our research, we tried to evaluate the abilities of the three indicators to exclude the possibility of sepsis and intracranial infection by ROC analysis. The AUCs for IL-2, IL-6 and IL-10 were 0.901 (95% CI, 0.866 to 0.930), 0.860 (95% CI, 0.821 to 0.894), 0.888 (95% CI, 0.851 to 0.918), respectively, indicating that IL-2, IL-6 and IL-10 were effective biomarkers to rule out sepsis and intracranial infection. The performances of IL-2, IL-6 and IL-10 to rule out the possibility of sepsis and intracranial infection are comparable with the role of CRP.

Lipoteichoic acid and lipopolysaccharide, at clinically relevant concentrations, induced differential cytokine/chemokine release in vitro and in vivo [25], [26]. Lipoteichoic acid is a cell wall component exclusive to Gram-positive bacteria and is shed during bacterial replication and after antibiotic administration [27]. While, lipopolysaccharide is the major cell wall component of Gram-negative bacteria [28]. So, we compared serum cytokine profiles when patients infected by Gram-positive bacteria or Gram-negative bacteria. Comparisons between gram-positive bacteria and gram-negative bacteria infection, IL-6 and IL-10 sharply elevated in patients with gram-negative bacteria infection (both P<0.001). In addition,IL-2 significantly decreased when patients suffered from Gram-negative bacteria infection (P = 0.031). Then, ROC curve was used to assess the diagnostic value of IL-2, IL-6, IL-10 and CRP to discriminate gram-negative from gram-positive bacteria infection by MedCalc 9.4.2.0 software. Using logistic regression to calculate predicted probabilities of various combination of cytokines, then the predicted probabilities were saved as a new indicator LOGREGR_Pred to assess the diagnostic value of different cytokines combinations by ROC curve. Our study shows combination of IL-2 and IL-10 (LOGREGR?Pred_IL-2+IL-10) is a more effective biomarker in predicting GNB infection. The specificity of the combination in prediction of gram-negative bacteria infection is better than CRP.

When compared with the classical enzyme-linked immunosorbent assay (ELISA), Flow cytometry-based inflammatory cytokine measurement is rapid and time effective. So, we suggest that quick measurement of cytokine profiles using the flow cytometry technique be transferred for clinical diagnostic purposes. Additionally, the day to day variance of the method is controllable and the technique is stable and the results obtained are consistent as long as we pay attention to the following key issues: careful daily maintenance of the flow cytometer; a qualified flow cytometry specialist with rich expertise; the standard curve is set up for each individual set of reagents and the blood serum is separated as rapidly as possible. On the other hand, since there are abundant assay kits available for serum cytokines detection and which are well correlated with ELISA [29], [30], we believe that these key findings in this study may also be valuable when translated to other comparable approaches.
